# The *Medicago truncatula *gene expression atlas web server

**DOI:** 10.1186/1471-2105-10-441

**Published:** 2009-12-22

**Authors:** Ji He, Vagner A Benedito, Mingyi Wang, Jeremy D Murray, Patrick X Zhao, Yuhong Tang, Michael K Udvardi

**Affiliations:** 1 Plant Biology Division, the Samuel Roberts Noble Foundation, 2510 Sam Noble Parkway, Ardmore, OK 73401, USA

## Abstract

**Background:**

Legumes (Leguminosae or Fabaceae) play a major role in agriculture. Transcriptomics studies in the model legume species, *Medicago truncatula*, are instrumental in helping to formulate hypotheses about the role of legume genes. With the rapid growth of publically available Affymetrix GeneChip Medicago Genome Array GeneChip data from a great range of tissues, cell types, growth conditions, and stress treatments, the legume research community desires an effective bioinformatics system to aid efforts to interpret the Medicago genome through functional genomics. We developed the *Medicago truncatula *Gene Expression Atlas (MtGEA) web server for this purpose.

**Description:**

The *Medicago truncatula *Gene Expression Atlas (MtGEA) web server is a centralized platform for analyzing the Medicago transcriptome. Currently, the web server hosts gene expression data from 156 Affymetrix GeneChip^® ^Medicago genome arrays in 64 different experiments, covering a broad range of developmental and environmental conditions. The server enables flexible, multifaceted analyses of transcript data and provides a range of additional information about genes, including different types of annotation and links to the genome sequence, which help users formulate hypotheses about gene function. Transcript data can be accessed using Affymetrix probe identification number, DNA sequence, gene name, functional description in natural language, GO and KEGG annotation terms, and InterPro domain number. Transcripts can also be discovered through co-expression or differential expression analysis. Flexible tools to select a subset of experiments and to visualize and compare expression profiles of multiple genes have been implemented. Data can be downloaded, in part or full, in a tabular form compatible with common analytical and visualization software. The web server will be updated on a regular basis to incorporate new gene expression data and genome annotation, and is accessible at: http://bioinfo.noble.org/gene-atlas/.

**Conclusions:**

The MtGEA web server has a well managed rich data set, and offers data retrieval and analysis tools provided in the web platform. It's proven to be a powerful resource for plant biologists to effectively and efficiently identify Medicago transcripts of interest from a multitude of aspects, formulate hypothesis about gene function, and overall interpret the Medicago genome from a systematic point of view.

## Background

Legumes (Leguminosae or Fabaceae) play a major role in agriculture the world over, accounting for one-third of the world's crop production. Seeds from legumes such as common bean, soybean, chickpea, and lentil are staple foods in many parts of the world and are important sources of protein, lipid, carbohydrate, and minerals while forage legumes such as alfalfa and clover are important sources of nutrition for livestock [1]. Legumes play a unique role in sustainable agriculture and in the global nitrogen cycle due to their ability to fix atmospheric nitrogen into organic form via symbiosis with rhizobial bacteria [2]. Symbiotic nitrogen fixation takes place in a specialized organ, the nodule, which develops from root cells following contact with rhizobia [3,4]. Legumes also form beneficial symbioses with soil fungi, which colonize root cells and transfer soil nutrients such as phosphorus to the plant [5]. As for all plants, legume growth and productivity are reduced by environmental stresses such as pathogens and pests, drought and salinity. Legume research is diverse and includes work on plant development, especially nodule and seed development, and plant responses to biotic and abiotic stresses.

Three legumes, *Lotus japonicus, Medicago truncatula*, and *Glycine max *(soybean) are the focus of current genome sequencing efforts [6-8], which will uncover most, if not all of the genes in these species. *M. truncatula *(or simply Medicago), like *L. japonicus*, was chosen as a model species for legume genetics and genomics because of its small diploid genome, self-fertility, short life cycle, high seed production, ease of cultivation and possibility of genetic transformation [9]. Soybean, although an ancient tetraploid with a genome twice the size of the other two legume species, was chosen for genome sequencing because of its economic value. With the impending completion of all three genomes, efforts to determine the function of many legume genes have come to the fore. Functional genomics is a new discipline that makes use of high-throughput transcript, protein, and metabolite profiling technologies, together with sophisticated tools and resources for reverse genetics, biochemistry, cell biology, and physiology to decipher the biological role of gene products.

Transcriptomics, the study of where, when, and to what extent genes are transcribed, is instrumental in helping to formulate hypotheses about the role of genes. A variety of massively-parallel measurement technologies, including arrays of gene-specific oligonucleotides for qPCR platforms [10,11] and RNA sequencing [12,13] enable quantification of transcript levels for most or all genes. The Affymetrix GeneChip^® ^Medicago Genome Array (abbreviated here to GeneChip) [14] contains probesets for the majority of Medicago genes and has become a popular tool for systematic study of the Medicago transcriptome. The Medicago Gene Expression Atlas (MtGEA) project, which initially provided gene expression data for the major organ systems of plants grown under ideal conditions and time-series for nodule and seed development, is based on the use of the Medicago GeneChip [15]. GeneChip data from a great range of tissues, cell types, growth conditions, and stress treatments have also been published recently [16-23].

To maximize the use of publicly-available Affymetrix GeneChip data and aid efforts to interpret the Medicago genome through functional genomics, we have developed the MtGEA web server, available at http://bioinfo.noble.org/gene-atlas/. This web server archives all publically-available *M. truncatula *gene expression data derived from the use of the Affymetrix GeneChip, and provides a range of web-based retrieval, analysis, and visualization functions for identification of genes of interest, exploration of their expression profiles, and prediction of their functions. In addition, all search results and the database as a whole, or part thereof, can be downloaded in tabular format to facilitate additional analysis by users.

## Construction and Content

**Figure 1:**
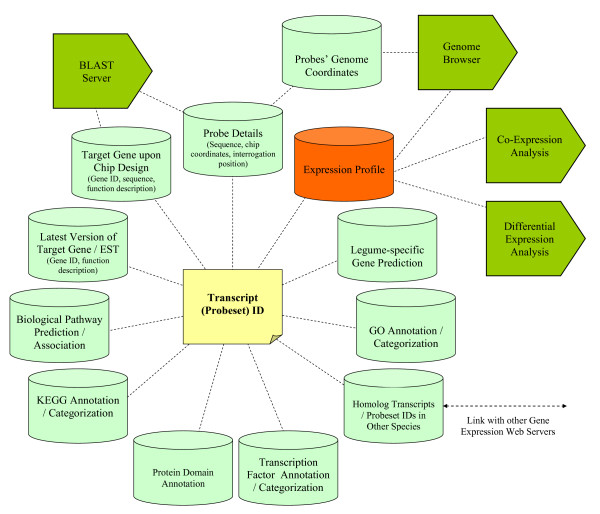
**Infrastructure of the *Medicago truncatula *Gene Expression Atlas web server**. Green database icons indicate different data types with corresponding search functions. Pentagons indicate the major analytical services provided by the web server.

The MtGEA web server contains a seamless fusion of multiple databases containing all publicly-available Medicago GeneChip expression data. Extensive annotation studies have been carried out for individual transcripts in order to predict their biological functions and their associations with the genome sequence. Figure 1 provides an overview of the different data types encompassed by the MtGEA web server, their interconnections with the implemented analysis tools, and the various search functions for data retrieval.

The MtGEA web server organizes information from heterogeneous sources into different database tables with clear separation for ease of maintenance. Tables are interconnected by common indexing of transcript (probeset) IDs to facilitate joint query of multiple databases. This physically-separate but logically-associated data organization style will facilitate future expansion of MtGEA with new datasets. Details about data acquisition, processing, and curation for all current data sources are provided below.

### Gene expression data

All publicly available *M. truncatula *gene expression data based on the Affymetrix GeneChip can be included in the MtGEA. So far, we have incorporated data from our own experiments and data published by other groups. MtGEA will be updated twice a year with newly available data.

The MtGEA web server currently hosts expression data from 64 experiments represented by 156 GeneChips [15-23] [Additional file 1: Supplemental Table S1]. We first collected the raw chip data in .CEL format for all samples: public data were downloaded from ArrayExpress [24]; our own experimental data were exported from the in-house GeneChip Operating Software (GCOS) [25]; and some data pending publication were directly obtained from our collaborators. In order to compare expression levels across all samples, all chips included in the database were then normalized together using the quantile method with Robust Multichip Average (RMA) [26]. The same set of .CEL files were also imported into the dCHIP software [27] to make presence/absence calls for each probeset using the software's default settings. The expression values, standard deviations, and presence/absence calls for each probeset were then combined and curated in the MtGEA web server.

It is noteworthy that the algorithm used to estimate the presence/absence calls in dCHIP is different from that in RMA. dCHIP's presence/absence call indicates if a gene is measured by the chip in a particular experiment; whereas RMA's call directly relates to the measurement level. The presence/absence calls in MtGEA are based on dCHIP's results as we believe they provide a better "on or off" indication about a gene's activities across multiple experiments.

The MtGEA web server organizes experiments according to genotype, organ/tissue/cell type, experimental factor (e.g. treatment), and data source. The annotation of the microarray samples was carried out manually through the data curation process, based on our careful review of the experiments' Minimum Information About a Microarray Experiment (MIAME) metadata and their corresponding publications. The annotative vocabulary was chosen according to terminologies commonly used by the plant biology community. Through the "Microarray Sample Selection" function, users can customize their selection of experiments for data analysis, visualization, and export. The web server retains the user's preference settings for one week after the last visit.

### Gene annotation data

#### Target Gene Mapping

In order to link each probeset to its corresponding gene or transcript, it was necessary to match genes' sequences to the probe sequences from the GeneChip. This is important since a large quantity of gene sequences (genomic and expressed sequence tags (ESTs)) have become available since the GeneChip was first designed so many of the original tentative consensus (TCs), singlet ESTs (singlets) and gene models used for the GeneChip design no longer exist. GeneChip probe sequences and their details (chip coordinates and interrogation positions), consensus sequences (the whole sequence of the transcript), target sequences (the region of the transcript sequence, or gene sequence from which unique probes were designed to compose the probeset) and their initial annotation were obtained from Affymetrix. Remapping of chip probesets to the latest version of International Medicago Genome Annotation Group (IMGAG, version 2) gene predictions and EST sequences (Medicago Gene Index; MTGI release 9) was done using a script developed by Affymetrix.

#### Homolog Transcripts

Medicago GeneChip target sequences were aligned with the target sequences of *Glycine max *and *Lotus japonicus *GeneChips using TBLASTX [28] searches with an E-value threshold set at 1.0e-5. The top five homolog transcripts from each species were indexed by MtGEA. More species and inter-site links will be included in the near future.

#### GO Annotations

The MtGEA web server currently incorporates two sets of Gene Ontology (GO) annotations [29]. GeneChip consensus sequences were aligned to sequences in the GO database [30] using BLASTX with an E-value threshold of 1.0e-4 on the PLAN server [31,32]. GO terms of the top hits to query sequences were exported by PLAN in tabular format and used to annotate the corresponding transcripts. The MtGEA server also includes GO annotations performed by Dr. Debby Samac's group in 2009 [33], which were based on alignments of GeneChip consensus sequences to sequences in the MTGI (version 9), and to Arabidopsis sequences at TAIR (version 7).

#### Legume Specific Gene Prediction, KEGG Annotation, Protein Domains, and Biochemical Pathways

Legume-specific gene prediction results were based on our previous work [15]. KEGG [34] annotation of Medicago transcripts was downloaded from the GeneBins web site [35,36]. Protein domain information was based on InterProScan of IMGAG v.2 gene models. Finally, a list of genes that were predicted to encode enzymes of biochemical pathways [37] was incorporated.

#### Transcription Factor Prediction

The MtGEA server indexes 1,169 transcription factor (TF) genes belonging to 48 families, which were identified previously [38]. We identified another 129 putative novel TF genes from consensus sequences based on the presence of DNA binding domains in the predicted proteins. A further 180 putative TF genes from 32 families were identified based on similarity to Arabidopsis TF genes using reciprocal BLAST searches. Annotations for all 1,478 putative TF genes are available at the MtGEA server.

### Software implementation

The repository of all MtGEA data utilizes the open source MySQL server 5 [39], which is currently hosted on a Linux server operated with the Fedora Core 8 distribution [40]. Both phpMyAdmin [41] and the MySQL built-in command line clients were used to curate and manage the data repository.

**Figure 2:**
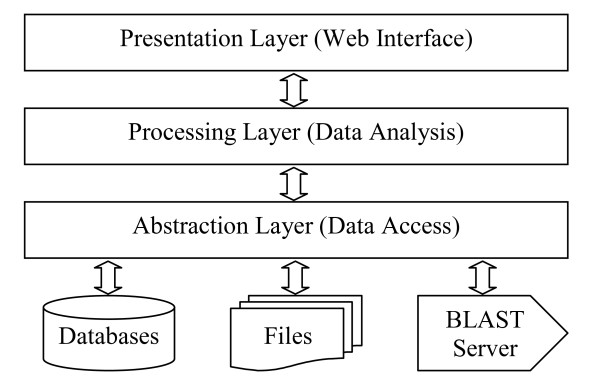
**Three-tier software architecture of the *Medicago truncatula *Gene Expression Atlas web server**.

As an integral component of the database, we have implemented a biologist-friendly web server to facilitate public access to the data. The web server follows a typical three-tier software architecture (Figure 2), which consists of a presentation layer for receiving user requests and rendering web pages, a processing layer for computation and data analysis, and an abstraction layer for data abstraction. The presentation layer was implemented with a collage of PHP, DHML and Java Script languages. A number of GNU web development packages including overLIB [42], Tab Pane [43] and Open Flash Chart [44] were used to build a highly interactive web interface. Cascading Style Sheets (CSS) and custom-designed web templates were adopted to generate uniform-looking web pages. The processing layer, programmed using PHP and Perl languages, contains various analytical modules for database search and result sorting, text string processing and numerical computation. Some BioPHP [45] and BioPerl [46] functions were utilized in the implementation. The abstraction layer, also implemented using PHP and Perl languages, contains a number of low-level data processing functions for file access, web session and program process management, as well as communications with the database server and the BLAST server. ADOdb [47] was adopted for database abstraction, which makes the system independent of the database server. These three tiers are logically defined and functionally integrated. It is worth mentioning that the use of platform-independent programming languages and database-independent abstraction layer makes MtGEA highly portable to other computer platforms and capable of handling gene expression data from other species with minimal additional effort on computer programming.

As one of the back-end services provided by MtGEA, the BLAST server was built using the NCBI BLAST toolkit [48]. In addition, an M. truncatula genome browser was built using the open source GBrowse viewer of the Generic Model Organism System Database (GMOD) [49], and was hosted on a dedicated server http://bioinfo4.noble.org/cgi-bin/gbrowse/gbrowse/medicago to be shared by multiple projects including MtGEA. The GBrowse web server in combination with a MySQL database server is used to store, search and display annotation of the M. truncatula genome. Necessary software customizations were done to ensure seamless interconnections between Mt genome browser and MtGEA.

## Utility and Discussion

Users can access the MtGEA web server at: http://bioinfo.noble.org/gene-atlas/. The server has been in operation since January 2008 and as of July 2009 has responded to over 15,000 data retrieval requests. The data retrieval and analysis options currently available are described below.

### Search by ID, key word or functional annotation

Users may provide one or multiple transcript (probeset) IDs (e.g. Mtr.26505.1.S1_at) to view and download associated features such as transcription profiles, annotations and genome coordinates. IMGAG v.2 gene IDs (e.g. AC145021_36.4) and MtGI TC IDs (e.g. TC108404) can also be used in this way. In addition, users can query the database using functional annotations with natural language (e.g. "ABC transporter"), one or multiple GO terms, KEGG terms, protein domain names, or through browsing of transcription factor families. For natural language queries, the server provides "exact match" and "approximately match" options.

### Search by sequence similarity

The MtGEA server offers sequence alignment via BLAST. It supports batch submission of multiple query sequences. Three BLAST databases are provided, namely the GeneChip consensus sequences, target sequences, and probe sequences. Users typically carry out a BLAST search against the consensus or the target sequence databases to identify Medicago sequences corresponding to the query sequences, and against the probe database to check if the query sequence can be measured by the probes present on the chip.

To help identify on the Medicago GeneChip potential homologs related to genes of other model legume species, MtGEA indexes the BLAST search results of target sequences relative to *G. max *(soybean) and *L. japonicus *probesets present on their respective GeneChips, thus enables the users to retrieve the homolog Medicago transcripts directly according to the GeneChip probeset IDs from these two species without carrying out time intensive BLAST search online. For example, users can enter a soybean probeset of interest and find the corresponding Medicago probesets.

### Search by genomic features

Through seamless inter-connections with the Medicago genome browser, users may investigate genomic features associated with genes of interest, including chromosomal position, IMGAG gene model structure, associated expressed sequence tags (ESTs), and the availability of flanking sequence tags (FSTs) in *Tnt*1 Medicago mutant collections [50]. As the consolidated genomic sequence becomes publicly available (IMGAG v.3), the information will be updated.

### Co-expression analysis and differential expression analysis

The MtGEA server enables batch retrieval of genes whose expression profiles are highly correlated to that of a chosen gene, through input of a probeset ID, or to a custom expression profile, through input of a numeric pattern. Users may customize the data points (corresponding to biological experiments/conditions) to be used for the correlation calculation. For example, the user may input a custom expression profile pattern "100 200 400 800 1600 3200" and choose samples "Seed10d Seed12d Seed16d Seed20d Seed24d Seed36d" to search for transcripts showing a defined pattern of gene expression during seed development. For each co-expression analysis session, users customize the co-expression calculation method (currently Pearson's Correlation Coefficient or Cosine Correlation [51]) and set a correlation threshold and the maximum number of transcripts to be returned.

The server provides a straight-forward, yet flexible fold-change computation module for differential expression analysis. The fold-changes of multiple data points over a reference sample with a custom threshold value, reflecting different degrees of either up-regulation or down-regulation, can be combined with logical "all" or "any" options for retrieval of genes of interest. For example, the user may search for transcripts that show at least two-fold changes in all (or any) of "Seed12d Seed16d Seed20d Seed24d Seed36d" samples over "Seed10d".

### Retention and organization of search results

We have implemented a comprehensive data archiving system to save all users' search results for up to seven days. Each search session is assigned a unique web address so that users may revisit their search and analytical results or share them with their colleagues (e.g. via email or through website address) without carrying out the same work again.

**Figure 3:**
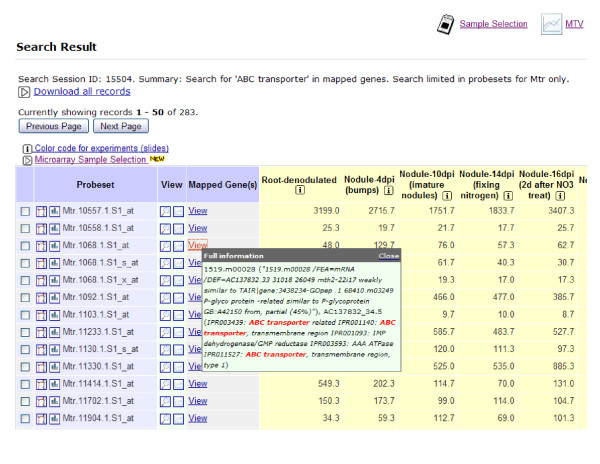
**A screenshot of the search result table summarizing each gene/transcript's major features**.

To allow compatibility with many data analysis applications, search results are summarized in tabular format and contain essential features of each transcript, including probeset ID, mapped genes/TCs with brief functional description, explanation of why this transcript is returned in the search session (e.g. with matching keywords or GO terms highlighted), a thumbnail-style visualization of its expression profile, and the expression profile in numerical form (Figure 3). By following a link provided in the search result spreadsheet, users may view further details of a transcript, including a customizable visualization of its expression profile, and related functional annotations and categorizations. This seamless integration enables users to perform in-depth investigation of the Medicago genome and transcriptome. For example, the user may browse all members of a particular transcription factor family, and then follow the links in the transcript details page to view their genome coordinates and corresponding gene models, and further search for co-expressed genes of each individual transcription factor and view the various functional annotations of these probesets.

**Figure 4:**
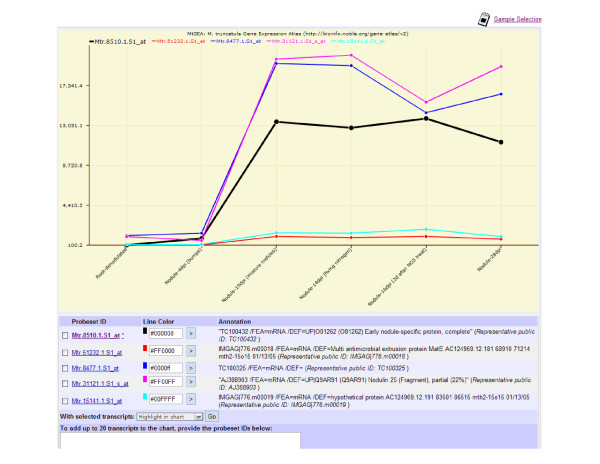
**An example of the Multiple-transcript Viewer chart that illustrates five co-expressed transcripts with the reference transcript highlighted**.

The MtGEA server we provides a "Microarray Sample Selection" function that allows users to limit their searches and analytical studies to a chosen subset of microarray experiments, and to customize the display of the search result table and gene expression profile plots. A "Multiple-transcript Viewer" is also provided to allow the users to visualize up to 20 gene expression profiles in a single graph, and to highlight one or multiple genes in the chart to aid comparison and improve presentation (Figure 4).

It is noteworthy that the MtGEA server provides a "Search/Analysis History" function that lists all searches and analyses conducted from the end-user's computer in the past seven days, and allows users to carry out logical combination of search results based on set operations ("UNION" or "INTERSECT", equivalent to "OR" or "AND" respectively). This enhances the flexibility and analytical power of the system. For example, a user may search for a particular term such as "kinase" in the functional description of transcripts, then carry out co-expression analysis against a reference gene expression profile for instance, expression only in nodules and flowers, and finally carry out an "INTERSECT" operation over these two search results to identify genes corresponding to kinases that are specifically expressed in nodules and flowers.

### Batch downloading data

All search results may be downloaded in batch through the search result summary page, where users may also customize the features of transcripts to be downloaded, e.g. annotation features, expression profile for selected experiments, whether to download single values of individual experiments or the mean of biological replicates, etc. The complete set of gene expression data, with natural language functional annotation, can be downloaded via the "Batch Download by Experiment" web page.

### Future development of the web server

It is intended that the MtGEA web server will be under continuous development to extend the range of data and processing options. Currently, we are working on the prediction and categorization of all Medicago transporters, which will be released in the near future. As soon as the consolidated IMGAG version 3 of the genome sequence is publicly released, we will update the probeset mapping and genome browsing features to offer users the most up-to-date dataset for analyses. We will also implement more differential expression analysis functions, and functions for programmable web access.

In the future, the web server will expand to encompass Affymetrix GeneChip data from other model legumes, such as *Lotus japonicus *and *Glycine max*, to provide an unparalleled resource for legume functional genomics.

Readers are encouraged to subscribe to our newsletter and read our News and Service Updates section to gain more information on our progress. Both options are available via our web server at http://bioinfo.noble.org/gene-atlas/.

## Conclusions

The MtGEA web server has a well managed rich data set, and offers data retrieval and analysis tools provided in the web platform. Its utility to the Medicago community during the past 1.5 years is reflected in the high volume of users who can effectively and efficiently identify transcripts of interest from a multitude of aspects, formulate hypothesis about gene function, and overall interpret the Medicago genome from a systematic point of view. With our active expansion of gene expression data and analytical tools, we aim to make the MtGEA server the first "port of call" for legume researchers interested in genome-wide expression data.

## Availability and Requirements

The MtGEA web server is publically accessible via http://bioinfo.noble.org/gene-atlas/. The web server is designed to be highly interactive. To take full advantage of the system, a user's web browser should support, and have the following features turned on: in-line frame, cookie, CSS, Java Script and flash. Most main stream web browsers for desktop computers (e.g. the latest versions of Internet Explorer, Firefox, Safari and Opera) currently support these features, whereas some web browsers for mobile devices (e.g. the PDA version of Internet Explorer) may not render all MtGEA server web pages correctly. Data downloaded from the MtGEA server are typically in tab-delimited ASCII (pure text) format and are supported by most text editor and analytical software (e.g. Microsoft Excel).

## Authors' contributions

All authors conceived the web server and scoped the development project. JH designed and implemented the web server. VB curated the majority of the data. MW deployed the Medicago genome browser and helped with data curation. JM helped with data curation, web server testing and improvement. PZ oversaw part of the bioinformatics work. YT processed the microarray data. MU oversaw the MtGEA project and helped to finalize the manuscript. All authors read and approved the final manuscript.

## Supplementary Material

Additional file 1 **Supplemental Table S1**. Experiments currently included in MtGEA. Microarrays are only included when meeting minimum criteria of experimental design, sufficient description and data quality. Click here for file
