# Supplementary material for: The Medicago truncatula gene expression atlas web server
Source: BMC Bioinformatics. 2009 Dec 22;10:441. doi: 10.1186/1471-2105-10-441 (PMC2804685; doi:10.1186/1471-2105-10-441)
Supplement: Additional file 1 — Supplemental Table S1. Experiments currently included in MtGEA. Microarrays are only included when meeting minimum criteria of experimental design, sufficient description and data quality. [file 1471-2105-10-441-S1.DOC]

| **Reference** | **Experiment** | **Organ** | **Treatment** | **Genotype** | **Culture System** | **Sample ID in MtGEA** | **Number of Samples** | **Number of Replicates** | **Number of GeneChips** |
| --- | --- | --- | --- | --- | --- | --- | --- | --- | --- |
| Benedito et al. (2008) | Mature whole organs (reference for optimal growth conditions) | Leaf, Petiole, Stem, Vegetative Bud, Flower, Pod, Root, Nodule | Reproductive organs and 28-day mature vegetative organs grown under optimal conditions (with exception of nodules, organs are from non-inoculated plants) | Jemalong A17 | Soil | leaf, petiole, stem, vegetative bud, stem, flower, pod, root, nodule | 9 | 3 | 27 |
| Benedito et al. (2008) | Nodule development series | Root, Nodule | Time course during nodule development after inoculation with rhizobia | Jemalong A17 | Aeroponics | Root-0dpi, Nodule-4dpi, Nodule-10dpi, Nodule-14dpi, Nodule-16dpi+NO3 | 5 | 3 | 15 |
| Benedito et al. (2008) | Seed development series | Developing seeds | Time course during seed development covering several stages after pollination | Jemalong A17 | Soil | Seed-10, Seed-12dap, Seed-16, Seed-20, Seed-24, Seed-36 | 6 | 3 | 18 |
| Naoumkina et al. (2007) | Challenged cell suspension cultures | Cell suspension | Temporal responses (0hy, 2h, 24h) of cell cultures with methyl-jasmonate and yeast elicitor | Jemalong A17 | tissue culture | CS_MJ_2HR_CK, CS_MJ_2HR, CS_MJ_24HR_CK, CS_MJ_24HR, CS_YE_0HR_CK, CS_YE_2HR-CK, CS_YE_2HR, CS_YE_24HR-CK, CS_YE_24HR | 9 | 2 | 18 |
| Imin et al. (2008) | Leaves from embryogenic and non-embryogenic genotypes under hormonal induction | Leaf | Genotypical and hormonal comparison of leaf cultures onto media supplemented with hormones | 2HA and Jemalong | Tissue culture | LF_2HA_1wk, LF_2HA_1wk_NAA, LF_2HA_2wks_NAA_BAP, LF_Jemalong_2wks_NAA_BAP | 4 | 3 | 12 |
| Holmes et al. (2008) | Spatial resolution of root tip | Root | 3-day-old root tip (3mm meristem) and 1-cm segment adjacent from root tip | Jemalong A17 | Tissue culture | RT_1cm_adj_tip, RT_3mm_tip | 2 | 3 | 6 |
| Uppalapati et al. (2009) | Roots challenged to biotic stress | Root | Time-course of roots exposed to *Phymatotrichum* (root rot pathogen) | Jemalong A17 | Tissue culture | RT_CRR_0hpi, RT_CRR_72hpi, RT_CRR_96hpi | 3 | 3 | 9 |
| Gomez et al. (2009) | Roots inoculated with mycorrhiza | Root | 6wk-old uninoculated and inoculated roots | Jemalong A17 | Soil | RT_Myc_CK, RT_Myc_Inf | 2 | 3 | 6 |
| Pang et al. (2008) | Transgenic roots expressing the Arabidopsis TT2 gene | Root | Transgenic hairy roots | Jemalong A17 | Tissue culture | RT_transgenic_GUS_TT2_CK, RT_transgenic_TT2 | 2 | 2 | 4 |
| Pang et al. (2008) | Seed coat expression | Seed | Excised seed coat | Jemalong A17 | Soil | Seed_Coat | 1 | 3 | 3 |
| Naoumkina et al. (2008) | Transgenic seeds | Seed | 20-22-dap transgenic seeds expressing the guar mannan synthase gene | R108 | Soil | Seeds_transgenic_R108_emptyVec, Seeds_transgenic_R108_GuarMannSynt | 2 | 3 | 6 |
| Daofeng Li (unpublished) | Response of roots to salt stress | Root | 2wk-old plants under salt stress (200mM NaCl) from 0 to 24h | Jemalong A17 | Hydroponics | RT_2wks_Sdl_Hydroponic_200mM_NaCl_0hrs_1rep, RT_2wks_Sdl_Hydroponic_200mM_NaCl_1hrs_1rep, RT_2wks_Sdl_Hydroponic_200mM_NaCl_2hrs_1rep, RT_2wks_Sdl_Hydroponic_200mM_NaCl_5hrs_1rep, RT_2wks_Sdl_Hydroponic_200mM_NaCl_10hrs_1rep, RT_2wks_Sdl_Hydroponic_200mM_NaCl_24hrs_1rep | 6 | 1 | 6 |
| Ruffel et al. (2008) | Responses of shoots and roots under several conditons of nitrogen supplementation (form and quantities) | Whole shoot, Inoculate root, Non-inoculated root | Split-root system supplied with sufficient, insufficient or absent forms of nitrogen (N2, NO3 and NH4) | Jemalong A17 | Hydroponics | SHT_5WKS_SuffN2_N2PAT, SHT_5WKS_LimtN2_N2PAE, SHT_5WKS_SuffNH4_NH4PAT, SHT_5WKS_LimtNH4_NH4PAE, SHT_5WKS_SuffNO3_NO3PAT, SHT_5WKS_LimtNO3_NO3PAE, RT_Nod_5WKS_SuffN2_N2S, RT_Nod_5WKS_LimtN2_N2C, RT_5WKS_SuffNH4_NH4S, RT_5WKS_LimtNH4_NH4C, RT_5WKS_SuffNO3_NO3S, RT_5WKS_LimtNO3_NO3C, RT_5WKS_NoNO3_NO3ONE | 13 | 2 | 26 |
|  |  |  |  |  |  | **Total:** | **64** |  | **156** |

**Supplemental Table S1.** Experiments currently included in MtGEA. Microarrays are only included when meeting minimum criteria of experimental design, sufficient description and data quality.
